# Japanese Encephalitis in Assam, India: Need to Increase Healthcare Workers’ Understanding to Improve Health Care

**DOI:** 10.1371/journal.pone.0135767

**Published:** 2015-08-21

**Authors:** Akram Ahmad, Muhammad Umair Khan, Lakhya Jyoti Gogoi, Manabendra Kalita, Atul Prasad Sikdar, Sureshwar Pandey, Sameer Dhingra

**Affiliations:** 1 Department of Clinical Pharmacy, Faculty of Pharmaceutical Sciences, Kuala Lumpur, Malaysia; 2 Institutional Level Biotech Hub, Mangaldai College, Mangaldai, 784125, Assam, India; 3 Institutional Level Biotech Hub, Dakshin Kamrup College, Mirza, 781125, Assam, India; 4 Department of Chemistry, Mangaldai College, Mangaldai, 784125, Assam, India; 5 School of Pharmacy, Faculty of Medical Sciences, The University of the West Indies, St. Augustine, Eric Williams Medical Sciences Complex, Uriah Butler Highway, Champ Fleurs, Trinidad and Tobago; University of California San Diego, UNITED STATES

## Abstract

**Introduction:**

Japanese encephalitis (JE) is a major cause of high morbidity and mortality in several states across India. However, in 2014, a sharp rise was observed in the number of cases of JE in north-eastern Assam state, and 51% of the total cases of JE in India were reported from the Assam in the same year. In this regard, a study was conducted to evaluate the knowledge and attitudes of healthcare workers in Darrang, a district of Assam highly affected by JE.

**Methods:**

A cross sectional study was conducted for 2 months among HCWs in the major district hospital of Darrang, Assam. A pre-tested, self-administered questionnaire was used to collect data from the participants. Convenience sampling approach was used to collect data from different departments of the hospitals. Descriptive and logistic regression analyses were used to express the results.

**Results:**

The knowledge of HCWs regarding JE was poor with a mean knowledge score of 11.02±2.39 (out of 17), while their attitudes were positive with a mean attitudes score of 43.16± 2.47 (ranging from 13 to 52). Overall, 40.4% and 74.3% of participants demonstrated good knowledge and positive attitudes respectively. Cut-off score for good knowledge and positive attitudes toward JE was set as ≥12 and >40 respectively. Older participants (40–49 years) and experienced workers (>10 years) were significantly associated with good knowledge as compared to their referent group (p<0.05), while knowledge of nurses and other orderlies were significantly lower than physicians (p<0.01). Similar factors were associated with the positive attitudes of the participants with the exception of experience. Television was the major source of information regarding JE reported by HCWs (79%).

**Conclusion:**

Although the knowledge was not optimized, HCWs exhibited positive attitudes towards JE. Future research is required to design, implement and evaluate interventions to improve the knowledge of JE among HCWs.

## Introduction

Japanese encephalitis (JE) is a serious illness caused by the Japanese encephalitis virus. Specifically, it is caused by the mosquito borne JE flavivirus. JE is the leading cause of viral encephalitis and disability in Asia. Solomon and colleagues reported that approximately 30,000–50,000 cases of JE are reported each year with an estimation of about 10,000 deaths annually [1]. According to World Health Organization (WHO) report, approximately 3 billion people are living in countries, which are at risk of JE in Southeast Asia and Western Pacific [2]. Children under 15 years of age are the major target of this fatal disease. Researchers reported that in areas where JE is endemic, nearly all cases are reported in children less than 10 years of age. Of 50% children who survive this disease are affected by various physical, cognitive and psychiatric problems [3]. Most often, infection due to JE virus remains asymptomatic, as on an average 1 in 300 patients produce symptoms. Generally, symptoms start to appear after an incubation period of 6 to 14 days. Fever, muscle pain, headaches, abdominal pains with nausea and vomiting, confusion, seizures and paralysis are the common sign and symptoms of JE [4 **–** 6]. JE virus is maintained in a cycle between mosquitoes and hosts like pigs and/or birds. Transmission of JE virus is predominant in rural agricultural areas, often associated with rice cultivation and flooding irrigation (monsoon season). Therefore, outbreaks are mainly associated with the rainy period which falls in summer and fall season (June to September) in India [7].

In India, JE is considered as a serious pediatric problem as its epidemic has been reported from many parts of the country. Since the first outbreak of JE in 1955 in Tamil Nadu, the outbreak of the disease has been reported on a regular basis in India [8]. However, the epidemic of 2005 surpassed all the previous outbreaks in India. Northern India, mainly Uttar Pradesh, was majorly affected by JE in that particular year resulting in 6061 cases and 1500 deaths [9]. In recent years, a sharp surge has been observed in the number of cases of JE in north eastern Assam state. Researchers reported that a five-fold increase in number of cases from 2010 (n = 154) to 2014 (n = 744) is mainly because of the recent changes in climatic conditions, agricultural practices, socio-cultural behaviour, abundance of potential mosquito vectors, and amplifying hosts in Assam state[10]. With regards to these statistics, Assam is positioned as a vulnerable state for JE. In 2014, more than 51% of the total cases of JE were reported form Assam [11]. Darrang is one of the few districts of Assam which is majorly hit by deadly JE. This is mainly due to high prevalence of vector populations (mosquito) and their amplifying host (animals) in this district as compared to others. Moreover, Darrang is also one of the most flood affected areas of Assam. Hence flooding of paddy fields also makes this district vulnerable for JE because of the increase in mosquito population [12].

The healthcare workers (HCWs) are regarded as an important members of the society to combat the disease outbreaks like JE. These individuals are uniquely positioned to improve access to care, health-seeking behaviour, and healthy behaviour. Additionally, healthcare workers are important source of information to general public as they provide counselling and educate community about the disease and its prevention. Therefore, the knowledge and attitudes of HCWs are needed to be standardized as it will be critical in educating and protecting communities. In view of this, and due to scarcity of published data, we conducted this study to assess the knowledge and attitudes of HCWs about JE in the most affected region of Darrang.

## Methods

A cross sectional study was conducted among HCWs in civil hospital of Darrang district. This is the main referral hospital of the studied district. The hospital receives financial support from the state government, hence treatment of patients is free or very less amount is charged, which increases the admission of patients from nearby areas and referral from government dispensaries and private clinics. In studied hospital, all the patients first have to attend outpatients clinics and then if needed they are sent to inpatient department for further investigation and treatment. The study was conducted for the period of 2 months from December 2014 to January 2015 in which the HCWs (physicians, pharmacists, nurse, laboratory technician and other orderlies) working in the district hospital for on a full time basis were invited to participate in this study. The sample size was calculated on the basis of Raosoft calculator in which the population size was kept as 500, power as 80%, response distribution as 50%, while confidence interval and margin of error was set at 95% and 5% respectively [13].The generated sample size (n = 218) was adequately powered to estimate the process parameters. All the participants were briefed about the nature and the objectives of the research before requesting them for their voluntary participation in this study. A convenience sampling approach was adopted in which the respondents were recruited on ease of accessibility; however efforts were made to recruit participants from different departments of the hospital. During the study period, participants were approached on continuous basis until the required sample size was achieved.

A self-administered questionnaire was designed and used as an instrument to collect data from the participants. A thorough literature review was done initially by two of the authors and relevant research papers were shortlisted for further discussions among authors [7,9,11,14–17]. After all the selected papers were comprehensively reviewed by the authors, an initial draft of the questionnaire was designed. The questionnaire was then subjected to content validity and face validity. The draft was sent to 3 pharmacy and medical academicians responsible for delivering lectures relating to infectious diseases for their opinion on the contents of the questionnaire. The suggestions given by the expert were incorporated and the second version of the questionnaire was developed which then sent to a small sample of 15 HCWs, currently working in healthcare settings for their suggestions on making the questionnaire more brief and simple. These responses were not included in the final analysis. Necessary changes were incorporated and after a series of discussion among the authors and between authors and the experts. A final version of the questionnaire was then distributed to the study participants for data collection. The data was then subjected to Statistical Package for the Social Sciences (SPSS) for reliability coefficient. A Cronbach's alpha of 0.72 and 0.77 was computed for knowledge and attitude sections respectively.

The study instrument consisted of four sections. First section highlighted the demographic information like gender, age, profession and years of experience. Second section, comprised of 17 questions, evaluated the knowledge of participants about the JE. Questions on knowledge were used to assess general knowledge of participants towards JE and its components. It assessed information like awareness, causes, sign and symptoms, transmission, incubation period, diagnosis and its management. Third section assessed the attitudes of respondents about JE based on 13 questions. The last part explored the source of information about JE. The knowledge questions consisted of Yes/No response categories. Knowledge scores ranged from 0–17 and cut off level of <12 were set for poor knowledge and ≥12 for good knowledge. Attitude questions consisted of 4 point Likert scale of agreement. A score of 1 was given to strongly disagree, 2 to disagree, 3 agree and 4 to strongly agree. The scale measured attitude from maximum 52 to minimum 13. Scores of ≤ 40 were taken as negative attitude, > 40 as positive attitude. Questions on attitudes were used to assess perceptions and beliefs of HCWS towards JE and its measures.

The responses of the participants were statistically analysed by using SPSS v.20. Descriptive analysis was performed and the results were expressed in frequency and percentages. Logistic regression analysis was used to assess the association between independent variables (demographic characteristics) and dependent variables (Knowledge and attitudes).P-value of less than 0.05 was reported as statistically significant.

### Ethics statement

The study was approved by ethical committee of Joint Health Services, Darrang district, Assam. Participation of respondents was voluntary and their responses were dealt with high level of confidentiality and anonymity. Participants were briefed about the objectives of the study and they were explained that the completion of the questionnaire would be taken as their consent to participate in this study.

## Results

**Fig 1:**
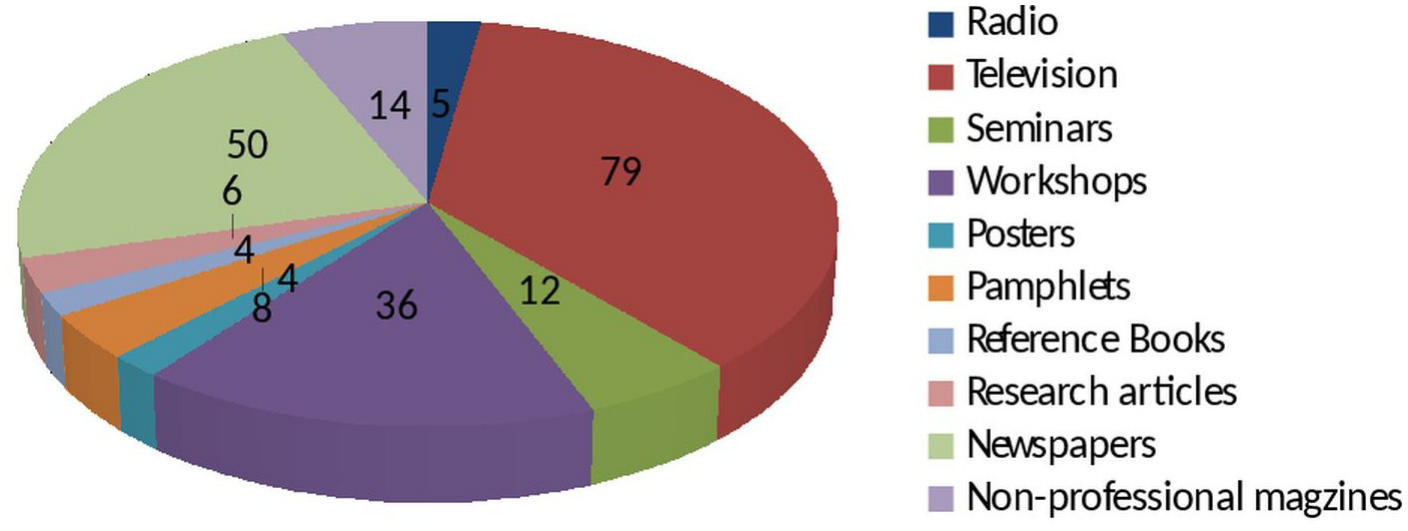
Sources of JE information reported by HCWs.

To recruit a sample of 218 participants, a total of 284 HCWs were randomly approached during a study period, giving a response rate of 76.7%. Majority of the participants aged less than 30 years (47.2%, n = 103), followed by 30–39 years (32.1%, n = 70) and 40–49 years (20.7%, n = 45) respectively. Laboratory staff formed the major proportion of the selected sample (32.5%, n = 71), while other orderlies were the least in numbers (5.6%, n = 12). HCWs with 3–6 years of experience were relatively large number in numbers (31.7%, n = 69) than those with 7–9 years of experience (17.4%, n = 38). The demographic information is summarized in Table 1. Television was the main source of information of HCWs about JE as depicted in Fig 1.

**Table 1:** Distribution of HCWs according to their characteristics (N = 218).

Characteristics	Healthcare Workers	
	No	%
**Gender**		
Male	141	64.7
Female	77	34.3
**Age in years**		
<30	103	47.2
30–39	70	32.1
40–49 ≥50	450	20.70
**Profession**		
Physician	49	22.5
Pharmacist	30	13.8
Nurse	56	25.6
Laboratory staff	71	32.5
Other orderlies	12	5.6
**Experience in years**		
<3	57	26.1
3–6	69	31.7
7–9	38	17.4
>10	54	24.8

The mean knowledge score of HCWs about JE was 11.02±2.39 (out of 17). Overall, 40.4% (n = 88) participants exhibited good knowledge of JE. HCWs were highly knowledgeable about JE (98.2%), its mode of transmission (97.7%), symptoms (92.7), prevention (93.1%) and its fatal consequences (94.9%).However, a large proportion of participants (89%) were not aware of the seasonal abundance and potential of JE. The numbers of incorrect answers were also high when participants were asked about the management of JE. 70.7% respondents incorrectly answered that antibiotics are the first line treatment of JE. Similarly, participants falsely answered that antivirals are highly effective against JE (67.4%). The results were not much different when HCWs were asked about the precautions need to be taken when dealing with JE patients as 63.3% respondents incorrectly replied to this question. The complete response of HCWs towards knowledge questions are presented in Table 2.

**Table 2:** Knowledge of HCWs towards Japanese Encephalitis (N = 218).

Questions	Responses			
	Incorrect		Correct	
	N	%	N	%
Have you heard about Japanese encephalitis?	4	1.8	214	98.2
JE is caused by virus	112	51.3	106	48.7
Mode of transmission of infection	5	2.3	213	97.7
Awareness of hallmark symptoms of JE	16	7.3	202	92.7
Incubation period of JE virus	123	56.4	95	43.6
ELISA is the method of choice for diagnosis of JE provided samples are collected 3–5 days after the infection.	126	57.8	92	42.2
Risk of JE in healthcare workers	37	17	181	83
JE can be fatal	11	6.1	207	94.9
JE is endemic to Asian countries	67	30.8	151	69.2
Japanese encephalitis is seasonal in its occurrence	194	89	24	11
Japanese encephalitis have a specific treatment therapy	92	42.2	126	57.8
People in rural agricultural areas are more prone to the JE	35	16.1	183	83.9
Vaccines are available for prevention of JE	15	6.9	203	93.1
Vaccines are available for prevention of JE in India	15	6.9	203	93.1
Antibiotics are first line treatment	154	70.7	64	29.3
Antivirals are highly effective against JE	147	67.4	71	32.6
Knowledge of precautions need to be taken when dealing with JE patients	138	63.3	80	36.7

Note: Knowledge was assessed by giving 1 to correct answer and 0 to wrong answer. The scale measured knowledge of maximum 17 to minimum 0. Score of < 12 were taken as poor knowledge, while score of ≥ 12 as good knowledge. Mean knowledge score was 11.02 ± 2.39.

The association of demographic characteristics and mean knowledge score is expressed in Table 3. Although males showed lower knowledge than females (31.1% vs 44.7%), the results were not statistical significant (OR = 0.81, p>0.05). In contrast, age and experience of participants were significantly associated with their knowledge of JE. Older participants (40–49 years) were more likely to have good knowledge (68.3% vs 35%) than respondents with less than 30 years of age (OR = 3.04, p<0.05). Similarly, the knowledge of experienced HCWs (>10 years) was also higher than those with less than 3 years of experience (86.5% vs 35.8%, OR = 17.33, p<0.001). No significant difference was observed between the knowledge of pharmacists and laboratory staffs with physicians (p>0.05); however, knowledge of nurses (OR = 0.06, p<0.001) and other orderlies (OR = 0.01, p<0.001) was significantly lower than physicians.

**Table 3:** Association of demographic variable with the knowledge of participants towards Japanese Encephalitis (N = 218).

Variables	Knowledge (%)		OR (95% CI)	*p*- value
	Poor	Good		
**Gender**				
Female	55.3	44.7	Ref	
Male	68.9	31.1	0.81 (0.32–2.01)	0.812
**Age (years)**				
<30	65	35	Ref	
30–39	51	49	1.64 (0.18–2.26)	0.497
40–49	31.7	68.3	3.04 (0.002–0.82)	0.037
**Profession**				
Physician	24.5	75.5	Ref	
Pharmacist	30	60	0.18 (0.02–1.30)	0.09
Nurse	91.1	8.9	0.06 (0.01–0.34)	0.001
Laboratory staff	22.3	77.7	1.48 (0.35–6.27)	0.59
Other orderlies	100	0	0.01 (0.004–0.46)	<0.001
**Experience (years)**				
<3	64.2	35.8	Ref	
3–6	53.6	46.4	1.50 (0.48–4.65)	0.47
7–9	57	43	1.48 (0.09–2.46)	0.38
>10	13.5	86.5	17.33 (6.6–45.36)	0.001

Note: Overall predictive accuracy of the model is 84.2%. Omnibus tests of model coefficients: Chi-square value = 119.455, p<0.001. -2 Log Likelihood = 169.941, Nagelkerke R square = 0.576

The mean attitudes score of HCWs towards JE was 43.16±2.47 (ranging from 13 to 52). Overall, 74.3% (n = 162) participants displayed positive attitudes towards JE. The response of HCWs was mostly positive for all the attitude questions. However, the most negative attitude was observed when HCWs favoured intensive and emergency treatment for patients diagnosed with JE (62.4%). Likewise, 25.2% participants disagreed to the statement that residents of an area where viral transmission is high should be vaccinated. Moreover, slightly less than one quarter of the respondents (22.4%) agreed or strongly disagreed that protective equipments should be used by HCWs when dealing with JE patients. HCWs response towards attitudes questions is presented in Table 4.

**Table 4:** Attitude of HCWs towards Japanese Encephalitis (N = 218).

Statements	Responses* N (%)			
	SD	D	A	SA
JE a serious illness	0	0	12 (5.5)	206 (94.5)
HCWs are at risk of JE	0	6 (2.7)	145 (66.5)	67 (30.8)
JE is a preventable disease	0	0	27 (12.3)	191 (87.7)
Controlling the breeding places of mosquitoes a good strategy to prevent JE	0	1 (0.6)	106 (48.6)	111 (50.9)
Stagnant water around the houses, broken pots and bottles are breeding places of mosquitoes responsible for JE	0	2 (0.9)	100 (45.9)	116 (53.2)
Communities should actively participate in controlling the vectors of JE	0	2 (0.9)	105 (48.2)	111 (50.9)
Everyone residing in areas with intense JE viral transmission should be vaccinated	0	55 (25.2)	44 (20.2)	119 (54.6)
Special Caution must be taken when dealing with patients of JE	5 (2.4)	2 (0.9)	99 (45.4)	112 (51.3)
Transmission of JE infection can be prevented by using universal precautions given by CDC, WHO, Government of India etc.	5 (2.4)	0	89 (40.9)	124 (56.7)
Prevalence of JE can be reduced by active participation of health care worker in hospital infection control program	0	0	111 (50.9)	107 (49.1)
Intensive and emergency treatment should be given to diagnosed patients	44 (20.2)	38 (17.4)	90 (41.3)	46 (21.1)
Healthcare workers must acknowledge themselves with all the information about JE	0	8 (3.7)	159 (72.9)	51 (23.4)
Gowns, gloves, mask and googles must be used when dealing with JE patients	31 (14.2)	18 (8.2)	103 (47.3)	66 (30.3)

Note: Attitude was assessed by giving 1 to strongly disagree, 2 to disagree, 3 to agree, 4 to strongly agree. The scale measured attitude from maximum 52 to minimum 13. Scores of ≤ 40 were taken as negative attitude, > 40 as positive attitude. Mean attitude score was 43.16 ± 2.47.

* SD = strongly disagree, D = disagree, A = agree, SA = strongly agree

The association of demographic characteristics and mean attitudes scores is tabularized in Table 5. Like knowledge, gender difference did not affect the attitudes of HCWs towards JE (p>0.05), while old age participants had significantly positive attitudes than younger respondents (OR = 4.95, p<0.001). Not much difference was observed between the attitudes of physicians, pharmacists and laboratory staff, however, the attitudes of nurses were more negative than physicians (OR = 0.33, p<0.05). Similar results were obtained when attitudes of other orderlies were associated with the physicians (OR = 0.24, p<0.05).

**Table 5:** Association of demographic variable with the attitudes of participants towards Japanese Encephalitis (N = 218).

Variables	Attitudes (%)		OR (95% CI)	*p*- value
	Negative	Positive		
**Gender**				
Female	33.8	66.2	Ref	
Male	21.3	78.7	1.40 (0.68–2.85)	0.352
**Age (years)**				
<30	31.1	68.9	Ref	
30–39	22	78	1.02 (0.32–1.26)	0.892
40–49	1	91.7	4.95 (0.61–10.05)	<0.001
**Profession**				
Physician	16.3	83.7	Ref	
Pharmacist	33.3	66.7	0.57 (0.05–1.35)	0.112
Nurse	41.1	58.9		0.041
Laboratory staff	18.3	81.7	0.33 (0.07–1.48)	0.783
Other orderlies	44.4	55.6	0.93 (0.27–5.68)	0.038
			0.24 (0.11–3.46)	
**Experience (years)**				
<3	26.3	73.7	Ref	
3–6	23.3	76.7	0.43 (0.16–1.16)	0.096
7–9	21.1	78.9		0.947
>10	17.6	82.4	0.94 (0.19–2.55)	0.714
			0.73 (0.13–1.87)	

Note: Overall predictive accuracy of the model is 74.4%. Omnibus tests of model coefficients: Chi-square value = 22.5, p<0.05. -2 Log Likelihood = 222.012, Nagelkerke R square = 0.146

## Discussion

To the best of our knowledge till date, this is the first study which has evaluated the knowledge and attitudes of HCWs about JE in India, as well as globally. In relation to this, our findings would relate to viral or vector borne diseases like malaria, dengue fever and other related illnesses.

The results suggest that the overall knowledge of HCWs participated in this study was poor, especially about the management of JE, while their knowledge was relatively better in areas like awareness of JE, transmission, symptoms, and vaccines. Similar results were observed when knowledge about JE was evaluated among care givers in Shaanxi Province, China, where the same baseline results were reported by the researchers [14]. Regarding management, participants showed lack of knowledge about pharmacotherapy, as majority of participants considered antibiotics as first line treatment. The JE is a viral borne disease and there is no role of antibiotics in the management of JE and there is no specific antiviral medication available in the treatment of JE [15].These findings indicate the need to take essential measures to bridge this knowledge gap by implementing effective interventions such as intensification of educational program, focussing more on the management, which may form one arm of this approach. These strategies are also supported by previous researchers in their report on the knowledge of Ebola virus disease (EVD) among HCWS in 2014 [18]. Majority of the participants in this study wrongly answered that JE is not seasonal in its occurrence (89%). This possibly indicates the lack of literature reading habit or participation in workshops or symposia by HCWs [17]. Efforts should be made to address this issue by encouraging HCWs for continuous medical education. The results were not different when a research was carried out at the time of swine flu epidemic in Saudi Arabia [19]. There is a need to promote health research among healthcare workers in India as it may provide them important information about disease trends, risk factors and public health interventions. Arranging research seminars on outbreak of diseases and other health issues on a regular basis may aide in achieving the needful objectives. Researchers have also shown that participation in research activities may enhance the understanding of healthcare workers regarding different disease conditions [20].

Furthermore, knowledge of laboratory staff was slightly higher as compared to physician, though the difference was not significant. In contrast, superiority of physicians was evident over nurses and pharmacist in terms of knowledge about JE. The results are in accordance to other studies that showed high knowledge of Physicians in terms of epidemic diseases [21, 22]. This finding could be possibly explained by the current healthcare system in India where physicians are seen as more clinically oriented professional as compared to other team members because of their in depth clinical training and more opportunities for professional development [23]. Therefore, it is equally important to educate other healthcare workers and community residents about epidemic or seasonal diseases and their preventive measures like vaccinations and precautions to prevent breeding mosquito in their homes and nearer areas. Policymakers and other concerned authorities should take essential measures to ensure the participations of other healthcare workers in infection control programs.

Furthermore, it is noteworthy to mention that experienced HCWs (> 10 years) were more knowledgeable as compared to junior ones. The results are in line with another study which also reported the superior knowledge of experienced healthcare workers [24]. The possible reason of these findings could be due to administrative positions held by senior workers which allow them to participate in different educational forums, conferences and discussion panels. This may increase the overall knowledge of experience workers about healthcare issues associated with current epidemics. These findings are in accordance with a report which suggested that experience workers are more effective in dealing with patients in healthcare settings [25]. Our study suggests that junior healthcare workers should also be focused and educational program must be designed to target this group to increase their knowledge about JE.

The mean attitude of the participants was positive. The attitudes of the physicians were more positive as compared to nurses, while older participants also showed positive attitudes towards JE. The likely reason of these findings could be due tothe fact that physicians are more associated with patient’s clinical record, counselling of patient than other HCWs. We believe that these activities are vital to shape the attitudes towards any disease. Our findings indicate that age and profession were the common factors significantly associated with the knowledge and attitudes of HCWs. Efforts should be made to customize interventions to target HCWs who are more likely to have inadequate knowledge and negative attitudes towards JE. However, there is a need to further study the patterns of knowledge and the expressed attitudes of the HCWs, and their association with the practices of HCWS [26].

The strength of this study is that it has explored an area where not much research has been done. Additionally, this study would help the stakeholders in India to design customized interventions to optimize the knowledge and attitudes of HCWs towards JE. However, this study also has some limitations like any other study. The results should be interpreted with cautions as the convenience sampling approach utilized in this single centre study may not be generalizable to the whole state or country. Additionally, we cannot ignore the tendency of participants to provide more socially desirable responses. However, despite of these limitations, our findings address a major healthcare problem that may confront India in future.

## Conclusion

The knowledge of HCWs towards JE was not optimized, however their attitudes were positive towards JE. Physicians and older participants were significantly associated with good knowledge and positive attitudes. Further studies are warranted to establish these results by including other major referrals hospitals of the affected states of India. Our findings could become a basis for the development of educational campaigns by targeting less knowledgeable areas highlighted in this study.
