# Correction: Japanese Encephalitis in Assam, India: Need to Increase Healthcare Workers' Understanding to Improve Health Care

**DOI:** 10.1371/journal.pone.0139648

**Published:** 2015-09-25

**Authors:** Akram Ahmad, Muhammad Umair Khan, Lakhya Jyoti Gogoi, Manabendra Kalita, Atul Prasad Sikdar, Sureshwar Pandey, Sameer Dhingra

There is an error in affiliation #1 for authors Akram Ahmad and Muhammad Umair Khan. Affiliation #1 should be: Department of Clinical Pharmacy, Faculty of Pharmaceutical Sciences, UCSI University, Kuala Lumpur, Malaysia.
